# Supplementary material for: Phylogeny and Flow Cytometry of the Genus Kalidium Moq. (Amaranthaceae s.l.) in Kazakhstan
Source: Plants (Basel). 2023 Jul 11;12(14):2619. doi: 10.3390/plants12142619 (PMC10383796; doi:10.3390/plants12142619)
Supplement: Supplementary file 1 [file plants-12-02619-s001.zip › plants-2425142-supplementary.pdf]

Supplements

Supplement A

Table -Origin, source, and GenBank accession numbers of *Kalidium* sequences made for phylogenetic analyses

Accession	Name	Coordinates	Voucher	rITS	trnQ-rps16	rpl32-trnL
B01	*Kalidium caspicum*	44.106944 N 67.056389 E	AA: 0001993	OQ061486	OQ077105	OQ077120
B03	*Kalidium caspicum*	45.875278 N 62.139167 E	AA: 0001995	OQ061487	OQ077106	OQ077121
B04	*Kalidium caspicum*	44.1775 N 78.812778 E	AA: 0001994	OQ061488	OQ077107	OQ077122
B07	*Kalidium caspicum*	45.848274 N 62.230855 E	AA: 0001992	OQ061489	OQ077108	OQ077123
B08	*Kalidium caspicum*	44.106111N 67.044444 E	AA: 0001998	OQ061490	OQ077109	OQ077124
B09	*Kalidium caspicum*	45.103361 N 64.531417 E	AA: 0002000	OQ061491	OQ077110	OQ077125
B10	*Kalidium caspicum*	44.080028 N 64.701056 E	AA: 0002001	OQ061492	OQ077111	OQ077126
B10.2	*Kalidium caspicum*	44.080028 N 64.701056 E	AA: 0002001	OQ061493	-	-
B11	*Kalidium caspicum*	44.109917 N 67.056361 E	AA: 0001999	OQ061494	OQ077112	OQ077127
B11.2	*Kalidium caspicum*	44.109917 N 67.056361 E	AA: 0001999	OQ061495	-	-
B12	*Kalidium caspicum*	43.680278 N 80.073333 E	AA: 0002002	OQ061496	OQ077113	OQ077128
B12.2	*Kalidium caspicum*	43.680278 N 80.073333 E	AA: 0002002	OQ061497	-	-
B14	*Kalidium caspicum*	43.759444 N 80.228889 E	AA: 0002003	OQ061498	OQ077114	OQ077129
B14.2	*Kalidium caspicum*	45.875278 N 62.139167 E	AA: 0002003	OQ061499	-	-
B14.3	*Kalidium caspicum*	45.875278 N 62.139167 E	AA: 0002003	OQ061500	-	-
B02	*Kalidium foliatum*	44.106944 N 67.056389 E	AA: 0001997	OQ061481	OQ077115	OQ077130
B05	*Kalidium foliatum*	44.1775 N 78.812778 E	AA: 0001996	OQ061482	OQ077116	OQ077131
B06	*Kalidium foliatum*	45.848274 N 62.230855 E	AA: 0001991	OQ061483	OQ077117	OQ077132
B13	*Kalidium foliatum*	43.680278 N 80.073333 E	AA: 0002004	OQ061484	OQ077118	OQ077133
B13.2	*Kalidium foliatum*	43.680278 N 80.073333 E	AA: 0002004	OQ061485	-	-
B15	*Kalidium schrenkianum*	43.759444 N 80.228889 E	AA: 0002005	OQ061501	OQ077119	OQ077134
B16	*Halocnemum strobilaceum*	43.796389 N 67.463611 E	AA: 0002038	OQ062601	OQ077135	OQ077162

B20	*Halostachys belangeriana*	44.106944 N 67.056389 E	AA: 0002031	OQ062605	OQ077138	OQ077165

Supplement B

Table - Sample data from NCBI database (only ITS)

Name	Locality	rITS
Kalidium caspicum	44.215833 N 86.655278 E	KX133017- KX133019
Kalidium caspicum	43.844444 N 90.624444 E	KX133020- KX133022
Kalidium caspicum	43.772500 N 91.721667 E	KX133023- KX133025
Kalidium caspicum	44.120556 N 87.714444 E	KX133026- KX133028
Kalidium cuspidatum	37.777672 N 95.292038 E	DQ340148
Kalidium cuspidatum	42.547884 N 93.981696 E	HM131637
Kalidium cuspidatum var. *cuspidatum*	36.708333 N 99.045833 E	KX133029-KX133031
Kalidium cuspidatum var. cuspidatum	37.344167 N 104.084722 E	KX133032-KX133034
Kalidium cuspidatum var. cuspidatum	37.669722N 107.515556 E	KX133035-KX133037
Kalidium cuspidatum var. cuspidatum	38.869722 N 106.756389 E	KX133038-KX133040
Kalidium cuspidatum var. cuspidatum	38.872778 N 108.698333 E	KX133041-KX133043
Kalidium cuspidatum var. *sinicium*	36.025278 N 97.647500 E	KX133044-KX133046
Kalidium cuspidatum var. sinicium	36.463333 N 103.932778 E	KX133047-KX133049
Kalidium cuspidatum var. sinicium	38.308889 N 103.275556 E	KX133050-KX133052
Kalidium cuspidatum var. sinicium	35.851389 N 94.520833 E	KX133053-KX133055
Kalidium cuspidatum var. sinicium	39.128889 N 100.551111 E	KX133056-KX133058
Kalidium foliatum	39.916111 N 105.705000 E	KX133059-KX133061
Kalidium foliatum	38.308889 N 103.275556 E	KX133062-KX133064
Kalidium foliatum	43.772500 N 91.721667 E	KX133065-KX133067
Kalidium foliatum	44.642778 N 83.252778 E	KX133068-KX133070
Kalidium foliatum	44.642778 N 85.268611 E	KX133071-KX133073
Kalidium foliatum	44.023813 N 113.898182 E	AY489238
Kalidium foliatum	47.612650 N 84.954706 E	DQ340150
Kalidium foliatum	46.658874 N 47.805919 E	KU975201
Kalidium foliatum	47.358223 N 47.358223 E	KU975200
Kalidium foliatum	47.189457 N 50.765061 E	AY181874
Kalidium foliatum	47.650950 N 87.775569 E	HM131638
Kalidium gracile	43.635000 N 91.957778 E	KX133074-KX133076
Kalidium gracile	38.853056 N 106.756389 E	KX133077-KX133079
Kalidium gracile	39.155278 N 98.170556 E	KX133080-KX133082
Kalidium gracile	40.845278 N 103.609167 E	KX133083-KX133085
Kalidium gracile	36.449167 N 103.984167 E	KX133086-KX133088
Kalidium gracile	44.755554 N 100.826111 E	DQ340151
Kalidium schrenkianum	41.925556 N 82.854167 E	KX133089-KX133091
Kalidium schrenkianum	42.113056 N 83.146667 E	KX133092-KX133094
Kalidium schrenkianum	41.590556 N 81.335556 E	KX133095-KX133098
Kalidium schrenkianum	45.833052 N 62.202842 E	KU975203

Kalidium wagenitzii	38.366888 N 33.564323 E	DQ340146

Note - the sample coordinates were taken from the articles

Supplement C

Matrix of SCoT primer results: SCoT 11 – 1-15; SCoT 12 – 16-33; SCoT 13 – 34-48; SCoT 14 – 49-64; SCoT 21 – 65-91; SCoT 23 – 92-109

B01.1_*K_caspicum*
1001000100110001000101100011100101100111001000000110100101000000001010010100000000010010011110000110110100000

B01.2_*K_caspicum*
1001000100110001000101000011000001000111000000000111000101010000000000010000000000010010011010000110100000000

B01.3_*K_caspicum*
1001000100110001000101000011000001100111000000000111000101010010000000010000000000010010000010000110100000010

B02.1_*K_foliatum*
0011000000110101000100000011011001100100001000110111110101011010000000011100001000000001101000000111000000010

B02.2_*K_foliatum*
0001000000110001000100000011000001101100000000010110000101000010000000011100001000000001101000000111000000000

B02.3_*K_foliatum*
0001000000110001000100000011000001101100000000010111000101000010000100011100001000000001101000000111000000000

B03.1_*K_caspicum*
0110100100110001001100000011100101100111100010000111000101000000101010010100011000100000111100101001010100101

B03.2_*K_caspicum*
0100100100110001000101000111000001100111100010000111110100010000101000010100110100100000000100001001000100111

B03.3_*K_caspicum*
0100100100110001000111100111000001100111100110000111100100010000101000010100110100100000000100001001000100111

B04.1_*K_caspicum*
10010001001100011001010010110001011001001100100001111101010100000001000110100000000000110011011000011000000010

B04.2_*K_caspicum*
100100010011000110011101001100000110010010110000011110010001000000010000101000010000001000100000000111000000000

B04.3_*K_caspicum*
10010001001100011001011010110001011001001100100001110001000100000001000110100000000000110011000100001110000010

B05.1_*K_foliatum*
00010000001100010011000000110000011101001000010001111100101101001100000101001010000000001100000000010110000010

B05.2_*K_foliatum*
00010000001100010001000000110000111001001000100001111101000100001100010101001010000000001100000000010100000010

B05.3_*K_foliatum*
00011000001110100001000000110000011001000000000001100001010000101000000001000001000010001000000000011100000010

B06.1_*K_foliatum*
1001100000111011000100100011010001101100000000010111010100011000000000011010001000000001101100001001100001010

B06.2_*K_foliatum*
1001100000111011100110000111010001101100000000010010000101000000010000011010001001100001101100001001101010010

B06.3_*K_foliatum*
10010000001100010101100001110100011011010000000101111101000110000100000110100010011000001
01100001001100001011
B07.1_*K_caspicum*
110000010011000100010110011110010110010110111000011111011001000000101001010000000000101000
01100001001100100111
B07.2_*K_caspicum*
110000010011000100010110011110010110010110111000011111011001000000101001010000000000101000
01100001001100100111
B07.3_*K_caspicum*
110000010011000100010110011110010110010110111000011111011001000000101001010000000000101000
01100001001100100111
B08.1_K_caspicum
110100010011000111111010111110010110011110101000011111110101000000101001010000000000001000
11100001011100000011
B08.2_*K_caspicum*
110100010011000111111010111110010110011110101000011111110101000000101001000000000000001000
11100001011100000011
B08.3_*K_caspicum*
110100010011000110111010101110010110011110101110011111110101000000101001010000000000001000
11100001011100000011
B09.1_*K_caspicum*
110100010011000000010000101100000110010010001000011111110101000000101001000000000000001100
11000001101000000010
B09.2_*K_caspicum*
110100010011000100110010101110000110010010001000011111110101000000101001010000000000001101
10000001001000000010
B09.3_*K_caspicum*
110100010001000100110010101100000110010010101000011111110101000000101001010000000000001100
11000001101000000010
B10.1_*K_caspicum*
110100010011000000010000001100000110010010101000011111111101000000101001010000000000001100
11100001011100000010
B10.2_*K_caspicum*
000100110011001100110010001100000110010010001000011101010101001010101001010010100000001001
00000110101000000000
B10.3_*K_caspicum*
100100010001000100110110001100000110010010001000011100010001001000101001000010100000001001
11100001111000000010
B11.1_*K_caspicum*
010100010001000100110000001100000110010010001000011111111101000000101001010000000000001000
11000010111000000000
B11.2_*K_caspicum*
000000010011001100010000001100000110010010001000011100011100001000101001010100110000001000
11000100001010000000
B11.3_*K_caspicum*
000000010011001100010000001100000110010010001000011101011101001000101001000000010000001000
11000000001000000000
B12.1_*K_caspicum*
100100010001101000010000001100000100010000000000011100010100001000101001010000000000001000
10000000001000000000
B12.2_*K_caspicum*
000001000001000100010110001100000110010010000000011111110101000000101001000000010000001000
10000000011000000000
B12.3_*K_caspicum*
100001010001000100011111001100010110010110000000011111110101000000101001010000100000001000
10100001010100000010
B13.1_*K_foliatum*

0011100000110011000110100011010001101100000000000111110101011000010000001010101000100001000000001001100000010
B13.2_*K_foliatum*
00010000001100000000100000000100000110010000000000011101101010000000100000011001010001000010
000000000001000000000
B13.3_*K_foliatum*
00011000001100110001001000110100011001001000000000111110101011000010000001010101000100000100000001000100000000
B14.1_*K_caspicum*
10111001010000111001011100110000011001111000000000111110101010000001010010100000000000010001010000100010000001
B14.2_*K_caspicum*
00010000101100011001001000110000011001001001110001111101010100001110010101000000001000100000100001000100000000
B14.3_*K_caspicum*
10111000100100011001010000110000011001001000000000111110101010000000010010100000000010010000010000100010000000
B15.1_*K_schrenkianum*
1001100010110101000101000011000001100100110000000110000101000001110100010100001001100010000000000001001000000
B15.2_*K_schrenkianum*
1001100010110001000110000011100001100100010000000110000101000001110100010100001011100010000000001001001000000
B15.3_*K_schrenkianum*
1001100010110001100101000011000001100100110000000110000100000001110100010010001001100010000000000001101000000

Supplement D. Interspecific location of the three species in the PCA (SPSS): A – Components 1 and 2; B – Components 1 and 3; C – Components 2 and 3

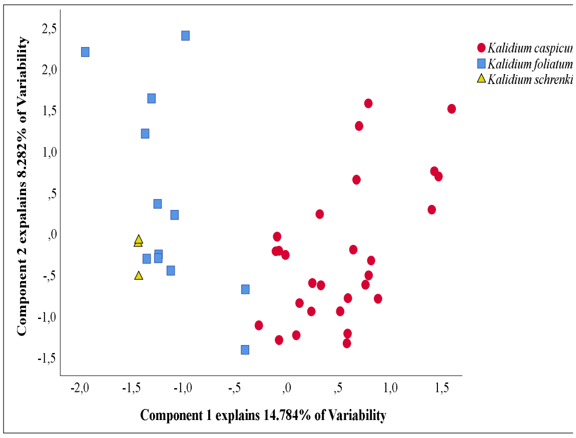

A

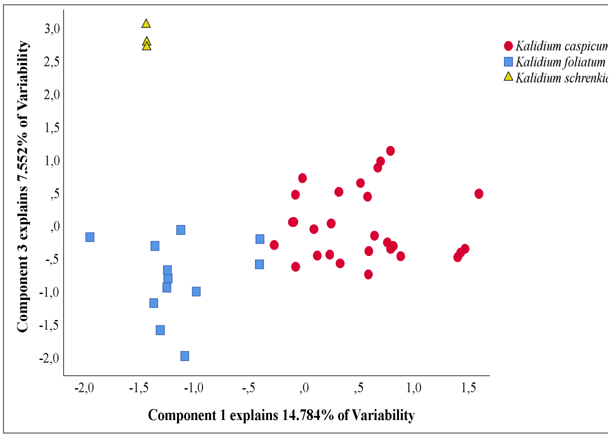

B

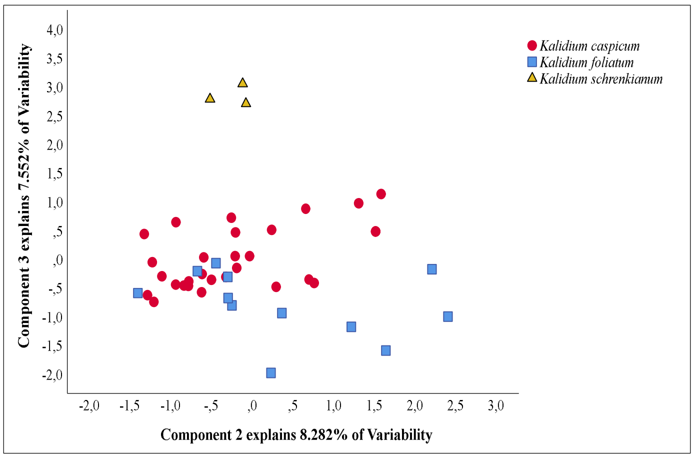

C

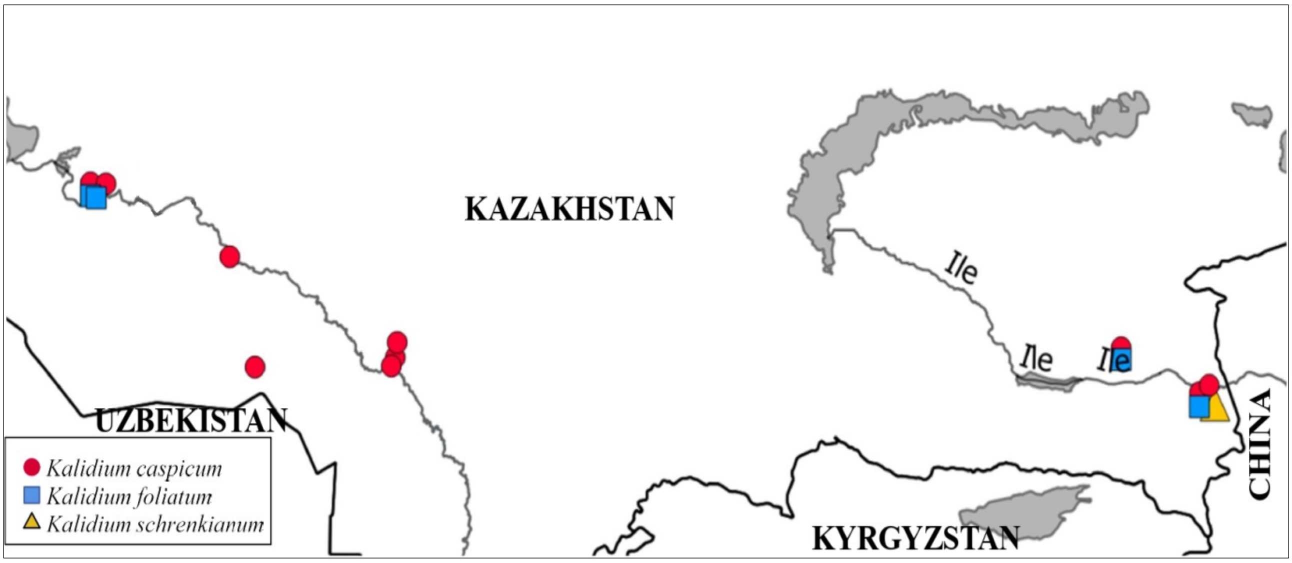

Map of the geographical distribution of the three species of the genus *Kalidium*

Supplement E. Intraspecific location of *Kalidium caspicum* populations in the histogram (SPSS): A – Components 1 and 2; B – Components 1 and 3; C – Components 2 and 3

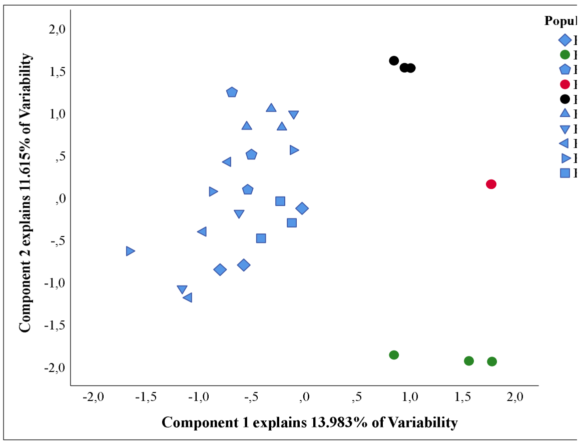

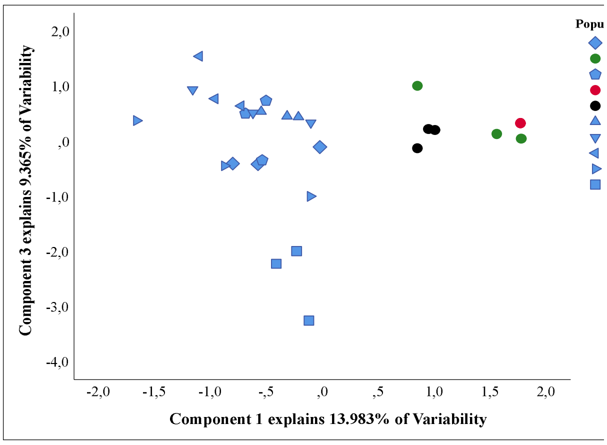

A

B

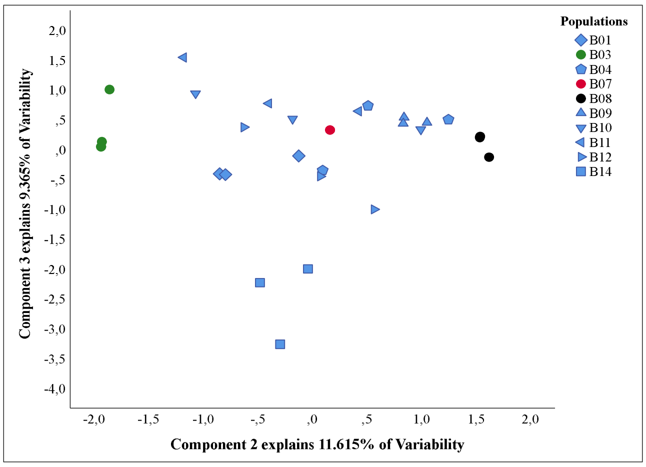

C

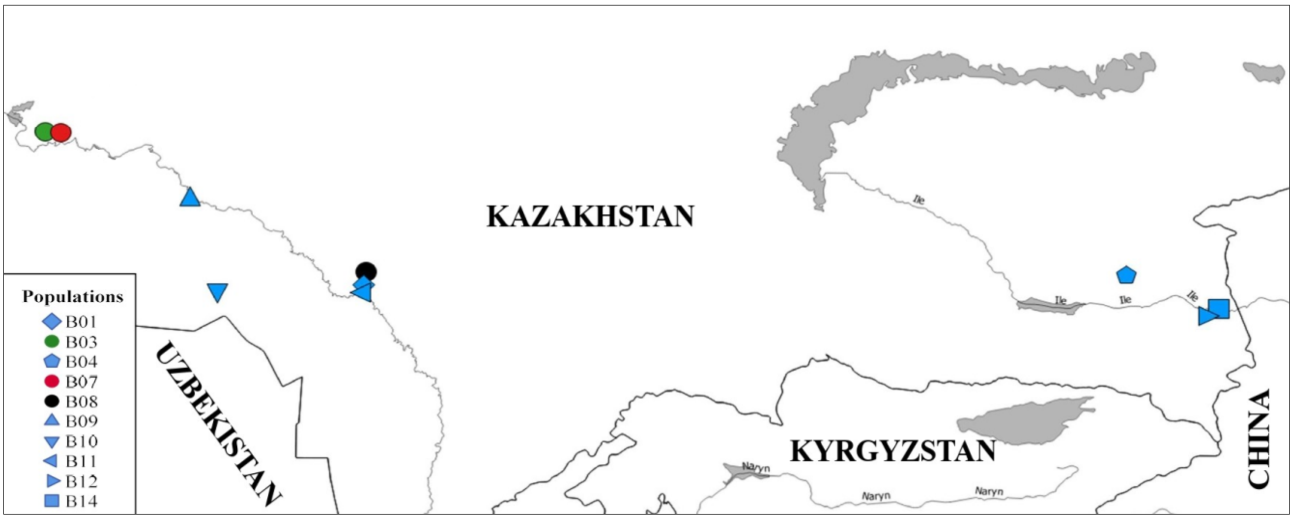

Map of the geographical location of *Kalidium caspicum* populations

Supplement F. Intraspecific location of *Kalidium foliatum* populations in the histogram (SPSS): A – Components 1 and 2; B – Components 1 and 3; C – Components 2 and 3

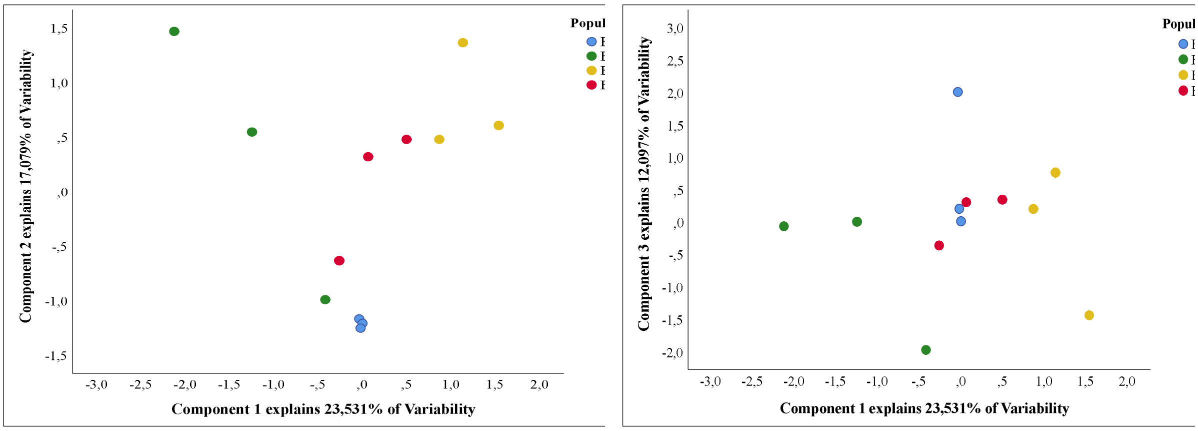

A B

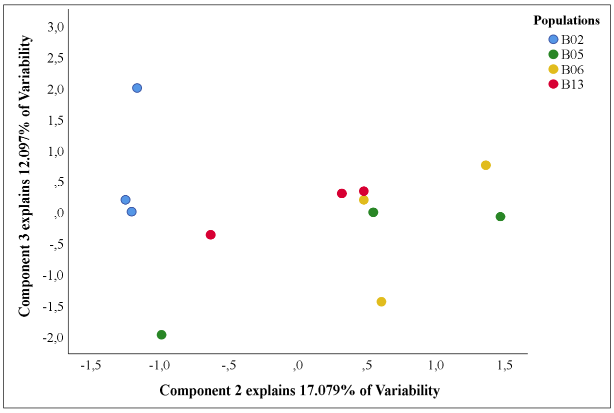

C

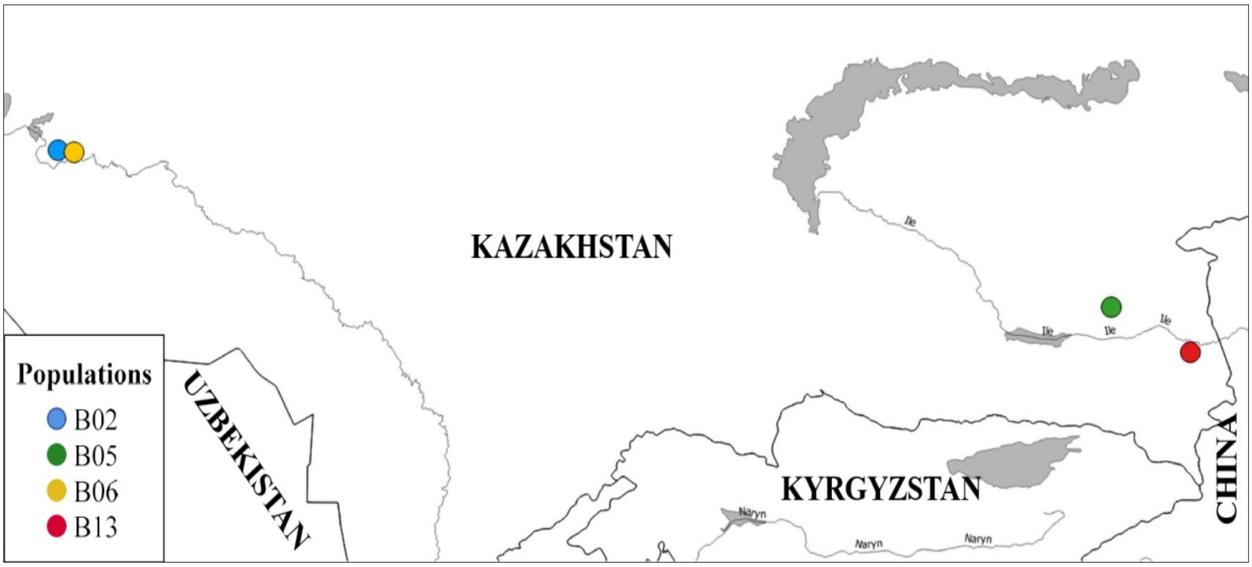

Map of the geographical location of *Kalidium foliatum* populations
